# High-throughput determination of enantiopurity in atroposelective synthesis of aryl triazoles†

**DOI:** 10.1039/d3sc01559a

**Published:** 2023-05-11

**Authors:** Jongdoo Lim, Melody Guo, Sooyun Choi, Scott J. Miller, Eric V. Anslyn

**Affiliations:** a Department of Chemistry, The University of Texas at Austin Austin Texas 78712 USA anslyn@austin.utexas.edu; b Department of Chemistry, Yale University New Haven Connecticut 06520-8107 USA scott.miller@yale.edu

## Abstract

Atropisomeric scaffolds are a common design element found in pharmaceuticals, many deriving from an N–C axis of chirality. The handedness associated with atropisomeric drugs is oftentimes crucial for their efficacy and/or safety. With the increased use of high-throughput screening (HTS) for drug discovery, the need for rapid enantiomeric excess (*ee*) analysis is needed to keep up with the fast workflow. Here, we describe a circular dichroism (CD) based assay that could be applied to the *ee* determination of N–C axially chiral triazole derivatives. Analytical samples for CD were prepared from crude mixtures by three sequential steps: liquid–liquid extraction (LLE), a wash-elute, and complexation with Cu(ii) triflate. The initial *ee* measurement of five samples of atropisomer 2 was conducted by the use of a CD spectropolarimeter with a 6-position cell changer, resulting in errors of less than 1% *ee*. High-throughput ee determination was performed on a CD plate reader using a 96-well plate. A total of 28 atropisomeric samples (14 for 2 and 14 for 3) were screened for *ee*. The CD readings were completed in 60 seconds with average absolute errors of ±7.2% and 5.7% *ee* for 2 and 3, respectively.

## Abstract 2

A facile, cost and time saving circular dichroism (CD) based assay is developed for high-throughput *ee* determination in atroposelective synthesis of aryl triazoles.

## Graphical abstract

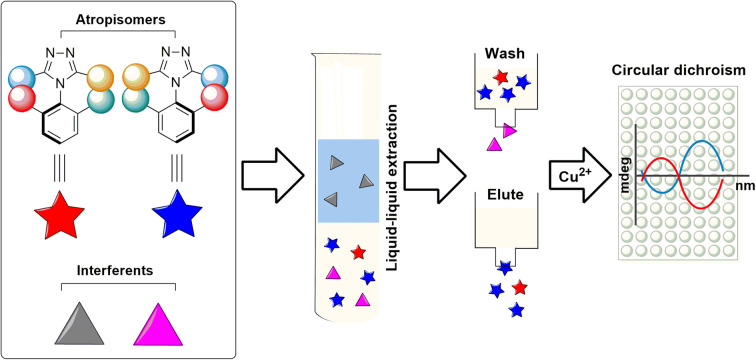

## Introduction

The therapeutic efficacy and toxicological profile of drugs are oftentimes influenced by their chirality because enantiomers can behave, interact, and bind differently to biological molecules such as nucleic acids, sugars, and proteins.^1^ From 2010 to 2020, drugs approved by the Food and Drug Administration (FDA) were predominately single enantiomers (approximately 60%), while only 3% of approved drugs were racemates.^2^ As a majority of pharmaceuticals marketed nowadays are semi- or fully-synthetic, asymmetric synthesis plays a key role in drug discovery. Such synthesis is largely conducted employing chiral auxiliaries and/or asymmetric catalysis. These reactions can be tuned to improve yield and enantiomeric purity by altering reaction parameters such as catalyst ligands, additives, solvent, temperature, and time.^3^ This approach to asymmetric synthesis generally relies on changing one parameter at a time, based on predicted reactivity and rational experimental design by human insight.^4^ With recent advances in automated parallel synthesis and combinatorial chemistry, an alternative has arisen – screening hundreds of experimental conditions in parallel, referred to as high-throughput experimentation (HTE).^5^

Because the chirality of target molecules is a pivotal characteristic affecting pharmacokinetic and pharmacodynamic profiles *in vivo*, the determination of enantiomer excess (*ee*) is one of the most important steps when new chiral drugs are evaluated. Chiral separation technologies such as chiral-phase high performance liquid chromatography (HPLC), chiral supercritical fluid chromatography (SFC), chiral gas chromatography (GC), and chiral capillary electrophoresis (CE) have been widely used for *ee* determination due to their good accuracy and precision.^6^ However, with a screening approach to reaction discovery, the number of analytical samples generated can be enormous, making the analytical assay the bottleneck for developing chiral small molecules. Because conventional chromatographic methods are inherently serial, they are not well-suited for HTE. Other chiral analysis alternatives include nuclear magnetic resonance spectroscopy (NMR),^7^ mass spectrometry (MS),^8^ and optical sensing.^9^ Optical sensing assays based on UV-Vis,^10^ fluorescence,^6,11^ and circular dichroism spectroscopy^12,13^ are gaining increased interest, as they can be performed in a parallel fashion with multi-well plates, allowing rapid *ee* determination at a low cost.^14,15^ These assays commonly rely on dynamic covalent assembly,^16–18^ click chiroptical sensing,^14,19,20^ and supramolecular systems (host-guest chemistry).^21–23^

**Fig. 1:**
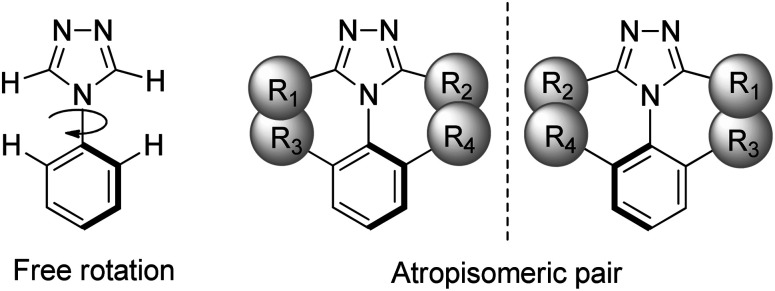
An atropisomeric aryl triazole pair arises when free rotation around the chiral axis is sterically hindered by bulky substituents.

Atropisomerism is a form of conformational chirality found in many substructures, such as biaryls, heterobiaryls, benzamides, and anilides.^24^ As shown in Fig. 1, atropisomerism is derived from hindered rotation along an axis of chirality due to bulky substituents. It is defined as a half-life for interconversion of the isomers >1000 seconds, being the time considered to be the minimum for separation and isolation, at a given temperature.^25^ About 15% of the existing small-molecule drugs approved by the FDA carry at least one atropisomeric axis, with an additional 10% of them being proatropisomeric.^26,27^

**Fig. 2:**
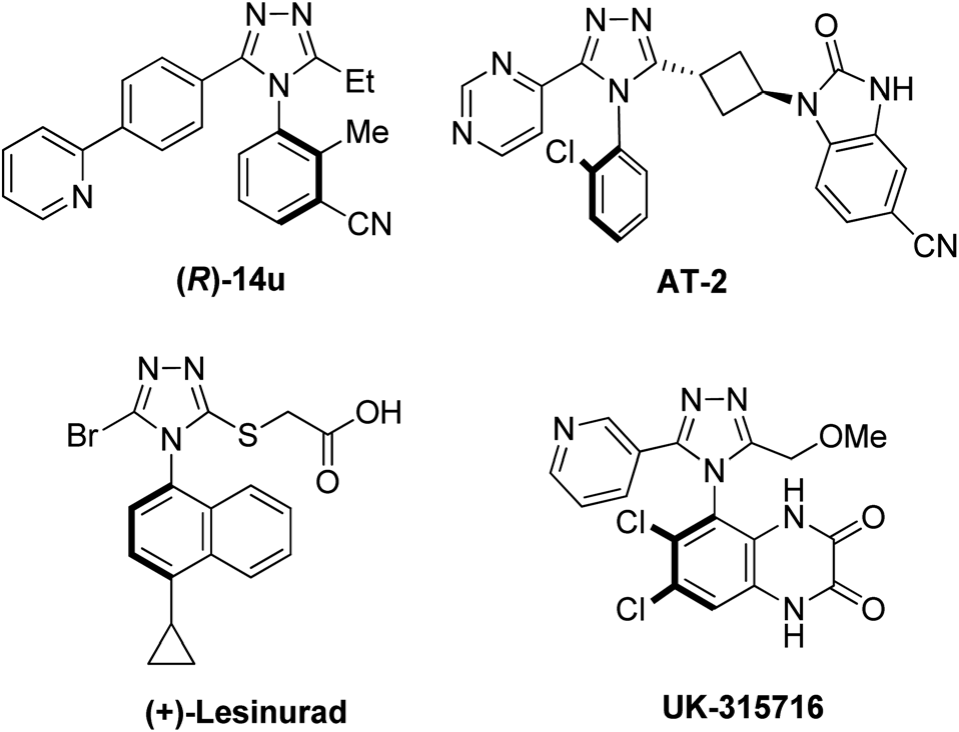
Atropisomeric *N*-aryl-1,2,4-triazole drugs.

*N*-Aryl-1,2,4-triazoles, and their derivatives, are important pharmacophores owing to their unique properties, including a large dipole moment, hydrogen bonding capability, and rigidity.^28^ They have a broad range of bioactivities: antiviral, antimicrobial, antitubercular, neuroprotectant, and anticancer.^29^ Oftentimes, the efficacy, selectivity, and pharmacokinetic behaviors of such triazole drugs are significantly influenced by their axial stereochemistry. For instance (Fig. 2), the (*R*)-isomer of the glycine transporter 1 inhibitor (14u) for rGlyT1 displayed an IC_50_ value of 0.064 μM, while the (*S*)-isomer showed a much lower activity (IC_50_ = 20 μM).^30^ Other atropisomeric triazole drugs have also been reported as having different potencies and activities depending on the axial stereochemistry. The IC_50_ values of (+)/(−)/(±)-lesinurad (hyperuricemia and gout treatment) are 4.4, 15.1, and 9.6 μM, respectively.^31^ The isomers (AT-1 and AT-2) of tankyrase inhibitor (an anticancer drug) showed a large difference in the IC_50_ values with a factor of 30 to almost 60.^32^ The atropisomeric quinoxalinedione, UK-31576 (stroke treatment), was developed by Pfizer as a single atropisomer.^33^ Thus, highly atroposelective synthetic methodologies are critical to modern drug discovery.

Generally, *N*-heteroatropisomers are synthesized by a chiral pool approach using chiral resolving agents, as well as an enantioselective synthetic approach utilizing the construction of a chiral C–N axis, the desymmetrization of prochiral/racemic *N*-heterocycles, or *de novo N*-heterocycle formation.^34–38^ To date, few optical sensing methodologies are available for determining the *ee* of *N*-heteroatropisomers.^39^ Thus, their enantiopurity is determined primarily by serial chromatographic methods.

**Fig. 3:**
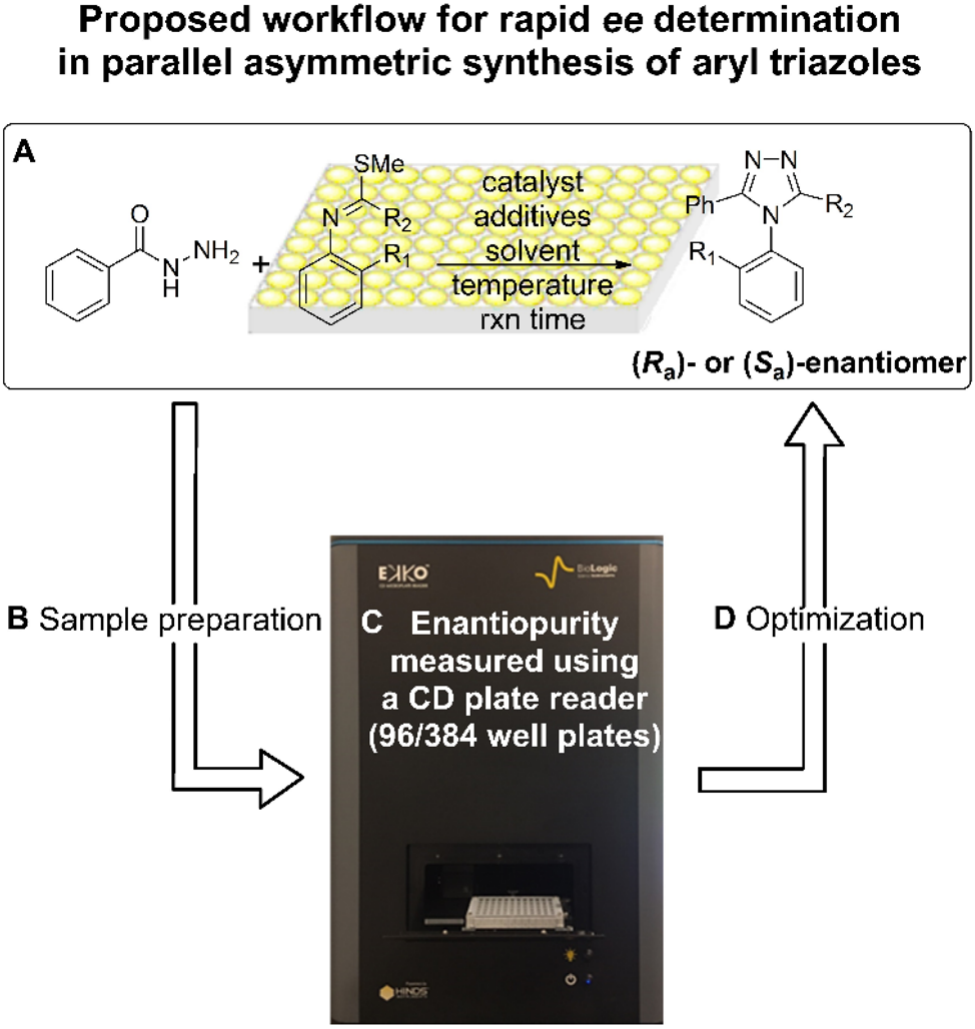
A schematic illustration of high-throughput *ee* determination for atropisomeric triazole products. (A) Parallel asymmetric synthesis of aryl triazoles *via* atroposelective cyclodehydration. (B) Analytical sample preparation. (C) High-throughput *ee* determination using a CD reader with a 96/384 well plate. (D) Optimization by altering reaction parameters.

Herein, we introduce an exemplar high-throughput platform for determining the *ee* values of atropisomers bearing C–N axial chirality, using simple metal coordination to generate CD-active species. As shown in Fig. 3, asymmetric synthesis of *N*-aryl-1,2,4-triazoles were performed *via* atroposelective cyclodehydration, followed by analytical sample preparation, analysed by a CD spectroscopy with a multi-well plate, and then optimized by altering reaction parameters. To be successful for high-throughput screening (HTS), the analytical workflow needs to be facile, fast, and parallel, with minimal purification steps. In addition, the CD signal and *ee* value should display a linear relationship. Lastly, automation needs to be amenable and inexpensive. We discuss the high-throughput *ee* determination of atropisomers of triazoles, addressing each of these challenges.

## Results and discussion

### Synthesis

**Scheme 1:**
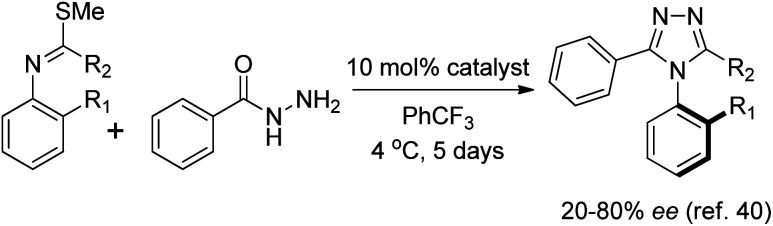
General route to atropisomeric triazoles.

The compounds utilized in this study were prepared using the method detailed in the manuscript by the Miller group (Scheme 1).^40^ The compounds were purified and concentrated after an extraction with saturated aqueous NaHCO_3_, or concentrated from the crude reaction mixture as needed. The triazole compounds used for this study were prepared with enantiomeric excess ranging from 20 to 80% *ee*, depending on the substrate and reaction parameters. The chiral phosphoric acid catalyst utilized could be appended to either a peptidic or a C_2_-symmetric BINOL-derived scaffold.^41^ These samples were then utilized for developing HT *ee* determination methods.

### CD enhancement with coordination chemistry

**Fig. 4:**
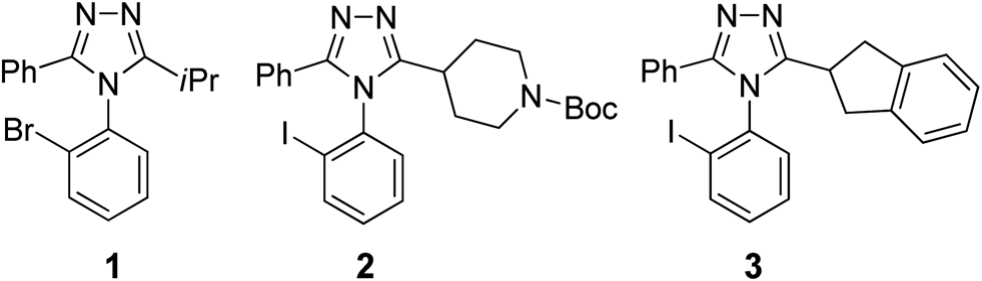
Three aryl triazole analytes (1, 2, and 3) were atroposelectively synthesized^40^ and employed for CD-based rapid *ee* determination.

With two or more proximal chromophores aligned asymmetrically, their transition dipoles can interact and couple to each other (exciton coupling), producing characteristic bisignate CD curves (Cotton effects). Such exciton-coupled circular dichroism (ECCD) is a highly sensitive and accurate spectroscopic tool to investigate the absolute configuration and conformation of chiral molecules in solution.^22,42,43^ The amplitude of ECCD is dependent on π–π* absorptions, interchromophoric distance, the angle of their twist, and the *λ*_max_ of chromophores.^44^ Accordingly, chiral molecules lacking suitable chromophores would display no or weak ECCD signals. Compounds 1, 2, and 3 are such structures, each having nearly no CD signals (Fig. 4). However, we envisioned using the ability to use triazoles as ligands to coordinate to metals, thus bringing two or more of them within proximity so that their chromophores would couple and lead to ECCD. Our approach was based upon the fact that the coordination chemistry of azoles is well-established and widely employed for catalysis, metal–organic frameworks (MOFs), and antifouling.^45–47^

**Fig. 5:**
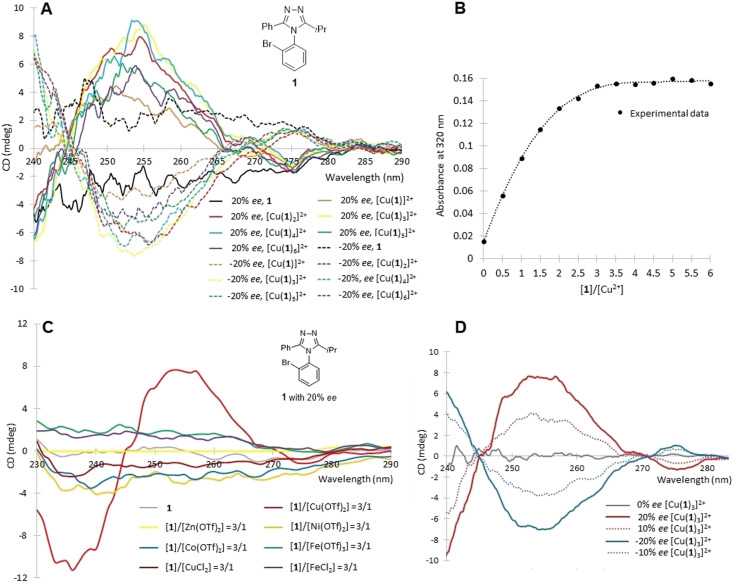
(A) The CD measurement of 1 (±20% *ee*) with addition of Cu(ii) triflate. The *y* over *x* in [Cu_*x*_(1)_*y*_]^2+^ indicate the ratio of moles present in solution, not the coordination number. (B) The titration of Cu^2+^ (fixed at 0.175 mM) by 1 in acetonitrile was performed at 320 nm (1 cm cell). (C) The CD spectra of 1 obtained with addition of various metal ions. (D) The CD spectra of [Cu(1)_3_]^2+^ with 0%, ±10%, ±20% *ee*, respectively. The CD spectra (A), (C) and (D) were recorded in acetonitrile at 20 °C (1.75 mM, 1 mm cell).

Initial coordination studies were conducted to determine a proper stoichiometric ratio of ligands-to-metal ions, and to identify a metal ion best suited to enhancement of ECCD. The three triazole atropisomers (1, 2, and 3) shown in Fig. 4 were examined. Potential CD-interfering molecules were also used to evaluate the necessity of purification. As the azoles are known ligands for Cu^2+^,^48^ solutions of 1 (±20% *ee*) in acetonitrile were prepared in varied ligand–metal molar ratios by addition of copper(ii) triflate, and analyzed by a CD spectroscopy (Fig. 5A). In Fig. 5, the *y* over *x* in [Cu_*x*_(1)_*y*_]^2+^ indicates the ratio of metal to ligand present in solution, not necessarily the actual coordination number. CD enhancement was observed upon addition of Cu^2+^. The amplitude of ECCD was maximized at [Cu(1)_3_]^2+^.

The UV-Vis titration of Cu^2+^ (fixed at 0.175 mM) by 1 in acetonitrile was performed at 320 nm where only the complexes, not the triazoles, absorb light (Fig. 5B). The plateau of titration curve occurred at a 3 : 1 binding stoichiometry (ML_3_), which either indicates three ligands per metal, or that the UV-Vis spectra does not change with additional ligands. This stoichiometry coincided with that found in the CD (Fig. 5A). Thus, CD spectra were recorded with complexes where the ratio of ligand-to-metal ratio was 3 : 1 (Fig. 5C). We next screened other transition metal ions for ECCD with the triazole ligands, as well as potential CD-active metal-to-ligand charge transfer (MLCT) bands in the longer wavelength region (>380 nm).^49^ With addition of the zinc, cobalt, nickel, or iron salts of trifluoromethanesulfonic acid, triazole 1 did not form ECCD-active complexes. Interestingly, different from Cu(OTf)_2_, CuCl_2_ also did not form a CD-active complex, presumably due to competition from chloride as a ligand. The CD of atropisomer 1 with 0, ±10, and ±20% *ee* was measured with complexation at a 1-to-Cu(OTf)_2_ molar ratio 3 : 1 (Fig. 5D). Albeit weak, the CD intensity displayed a linear relationship with *ee* despite the low enantiopurity (≤20% *ee*). These initial experiments utilized compounds with low enantiopurity due to the parallel nature of the synthetic and analytical developments for the title compounds. The complexation reactions reached equilibria immediately, which is advantageous for HTE.

### CD based assay for *ee* determination and validation study

**Fig. 6:**
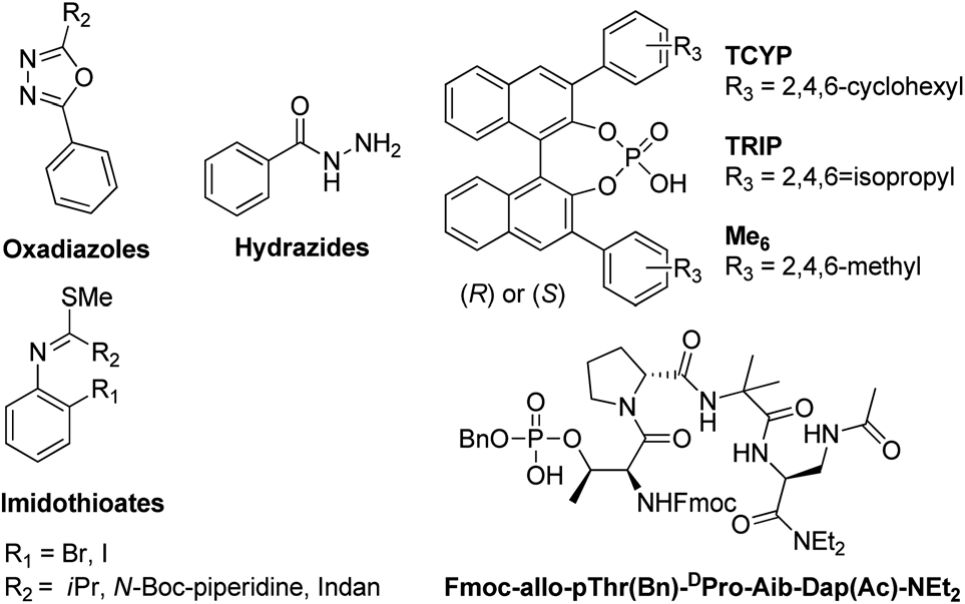
Potential interfering species for the CD based assay employed here: starting materials (hydrazides and imidothioates), by-products (oxadiazoles), and catalysts (TCYP, TRIP, Me_6_, or Fmoc-allo-pThr(Bn)-^D^Pro-Aib-Dap(Ac)-NEt_2_).

**Fig. 7:**
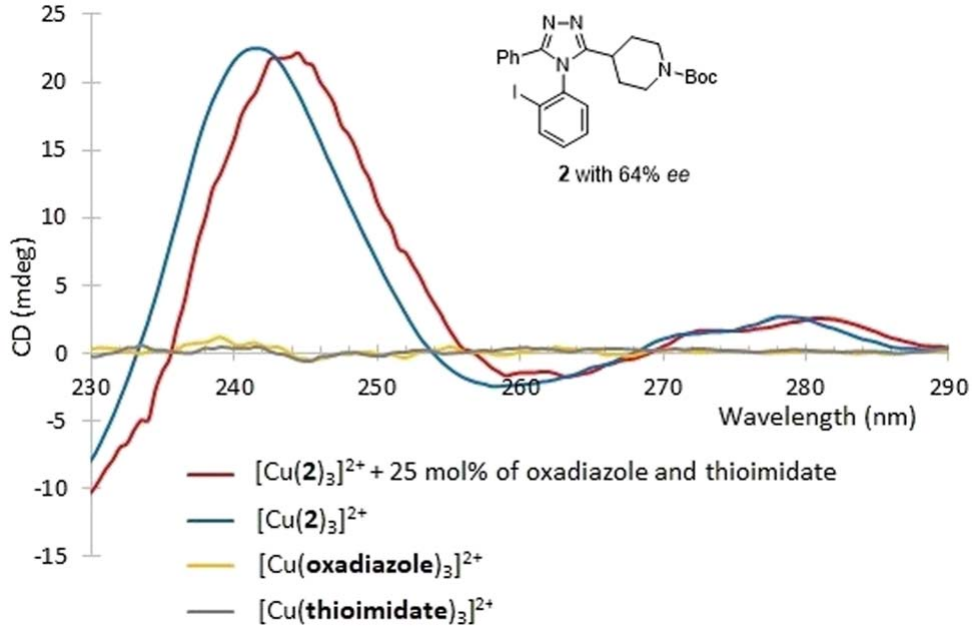
The CD assay was evaluated in the presence of two potential interfering species, the oxadiazole and the imidothioate.

The CD-active absorption bands of 1-Cu^2+^ complexes were observed in the UV region (*λ*_max_ = 243 nm). In this region, there are possible interferents resulting from the synthesis. Specifically, the two starting materials (the imidothioate and the hydrazide), the oxadiazole by-product, and the catalyst (Fig. 6); all of which are either chiral and thus CD active, or simply absorb UV light and thus affect the CD intensity. Among them, the catalyst and the hydrazide could be removed by simple liquid–liquid extraction (LLE). Therefore, as shown in Fig. 7, the CD assay employed here was first evaluated in the presence of two potential interfering species that could not be removed by LLE. Two solutions were prepared, one from pure 2 and a second as a mixture of 2 containing 25 mol% of the imidothioate and the oxadiazole at a 2-to-Cu(OTf)_2_ molar ratio 3 : 1. The CD spectra displayed a similar pattern, exhibiting the same CD intensity at 243 nm. This result was encouraging because a purification process could be unnecessary. However, the UV-absorbing interferents limited the concentration of analytical samples, reducing the intensity of CD signal. Thus, we decided to explore a simple and fast triage method that would be applicable to HTE. LLE was conducted using dichloromethane and 0.01 M HCl (aq.) as the two immiscible phases. The resulting extract was purified by a silica cartridge (HyperSep™, silica 50 mg), employing a two-step fast wash-elute procedure. A first washing was conducted using 1 : 3 or 1 : 4 ethyl acetate-hexanes. Second, the triazole product was then eluted using 7 : 1 dichloromethane-methanol. Such a purification protocol could work for other similar reactions that contain products and interferents differing in polarity, p*K*_a_, and aqueous solubility. In addition, this triage protocol can be conveyed to moderate-/high-throughput workstations with a 96-well format. Such workstations are commercially available and can execute the LLE and the wash-elute process in a parallel fashion, facilitating sample preparation for analysis.^50–52^

**Fig. 8:**
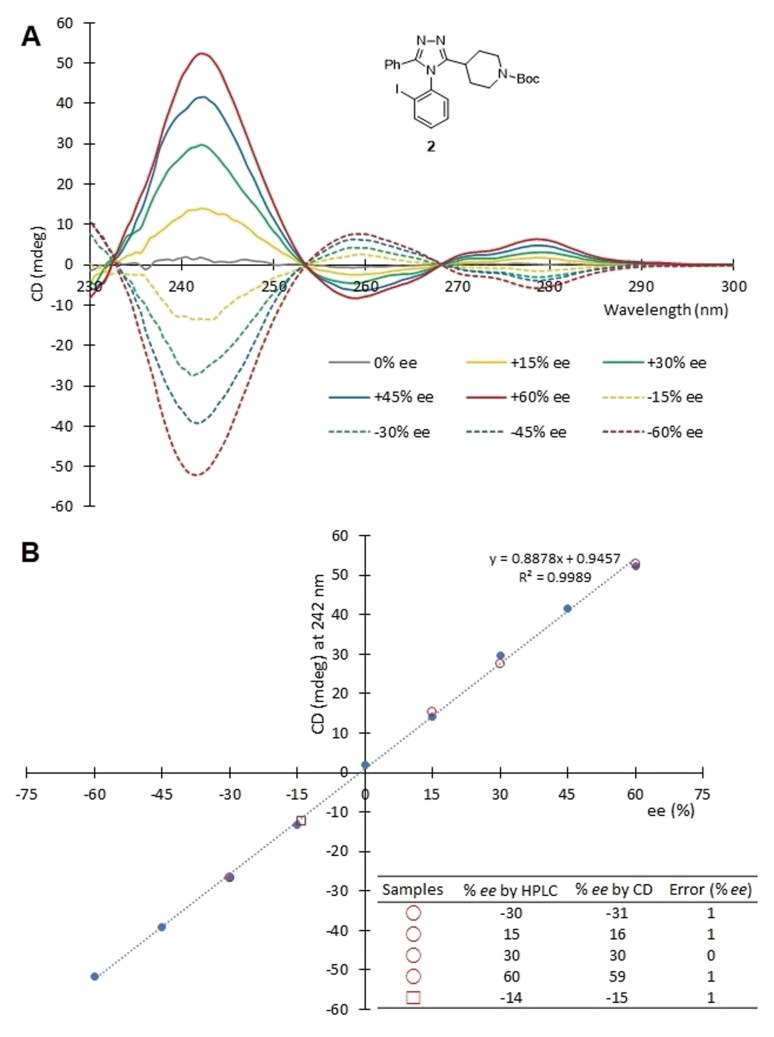
(A) CD spectra were recorded with [Cu(2)_3_]OTf_2_ having different *ee* values in acetonitrile. (B) A calibration curve was created and used to validate the CD assay employed here. Four samples were crude mixtures containing 2, the oxadiazole, and the imidothioate (red circle). One sample was an actual reaction mixture (red square). After these five samples were purified and complexed with Cu(OTf)_2_, their *ee* values were determined using the calibration curve.

First, the CD spectra of uncontaminated [Cu(2)_3_]^2+^ complexes with known *ee* values (0, ±15, ±30, ±45, ±60% *ee*) were obtained at a set concentration (1.75 mM, 1 mm cell) of 2 using a Jasco J-815 CD spectrometer. A calibration curve was constructed by plotting the ellipticity (*θ*) of complexes against the corresponding *ee* value of 2 at 242 nm. The calibration curve was found to be linear (*R*^2^ = 0.9989). Next, a validation of the CD assay for use with crude samples was performed using five samples containing atropisomer 2 with different *ee* values (Fig. 8). Four of the samples (red circles in Fig. 8B) were generated by mixing purified 2, purified oxadiazole, and purified imidothioate in the mole ratio of 1 : 0.25 : 0.25. One sample (a red square in Fig. 8B) was obtained from an actual reaction for the atroposelective synthesis of 2. This reaction used the peptide Fmoc-allo-pThr(Bn)-^D^Pro-Aib-Dap(Ac)-NEt_2_ as a catalyst, and the crude mixture was obtained after washing with saturated NaHCO_3_ and concentration. We employed the protocol discussed above that includes an LLE, a wash-elute process, and Cu^2+^ complexation. The *ee* values were determined by correlating the ellipticity of the samples to the calibration curve. The error in *ee* determination using the CD assay was ≤1% *ee* for all five samples, when compared to the *ee* values obtained by chiral HPLC (Fig. 8B). Hence, the assay adopting a simple triage protocol can be carried out with a low-level of error.

### High-throughput *ee* determination with a CD microplate reader

**Fig. 9:**
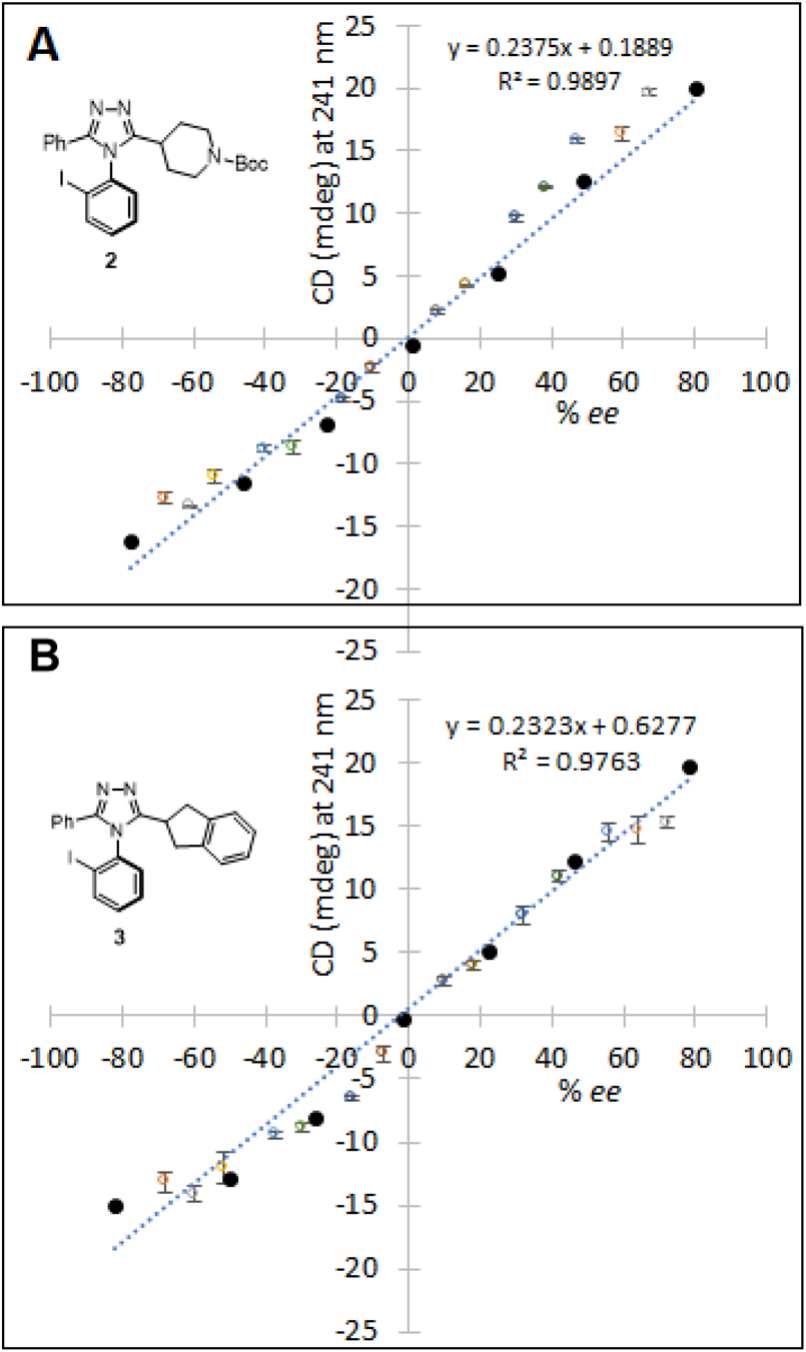
Calibration curves were constructed using the complexes of 2 and 3 with known *ee* values (●). The unknown *ee* values of samples were determined by correlating the ellipticity to the calibration curve. (A) Fourteen samples (○) of [Cu(2)_3_]^2+^ with unknown *ee* values. (B) Fourteen samples (○) of [Cu(3)_3_]^2+^ with unknown *ee* values.

With the encouraging results in the preliminary study, the high-throughput *ee* determination of 2 and 3 was performed by the use of a CD microplate reader (EKKO™ manufactured by Hinds Instruments). The CD reader has a capability to scan 96 samples in less than 2 minutes at a single wavelength, also providing an optional 384-well microplate format. In order to generate a sufficient number of samples with different *ee* values, atroposelective syntheses using (*R*)- or (*S*)-phosphoric acid catalyst TCYP were first conducted, yielding four enantioenriched reaction solutions: (*R*_a_)-rich 2, (*S*_a_)-rich 2, (*R*_a_)-rich 3, and (*S*_a_)-rich 3. The crude reaction solutions were concentrated directly from the reaction, redissolved in dichloromethane, and mixed in different ratios (v/v), generating a total of 28 (14 for 2 and 14 for 3) solutions. These solutions were utilized to validate the HT CD assay for determining *ee* (Fig. 9). Calibration curves for 2 and 3 were constructed, exhibiting *R*^2^ of 0.9897 and 0.9763, respectively.

The samples for CD-based analysis were prepared using the same method discussed: liquid–liquid extraction, a wash-elute process using a silica cartridge, followed by complexation with Cu(OTf)_2_. The detailed protocol of sample preparation is found in the ESI.† The ellipticity values of unknown samples were then recorded on the CD microplate reader. Subsequently, the *ee* values of samples were determined by correlating the ellipticity to the *ee* value *via* the calibration curve. The CD assay for HT *ee* determination was then validated against chiral HPLC. As shown in Table 1, the average differences between the CD and HPLC (% *ee*) in the HT *ee* determination of 2 and 3 were 7.2 and 5.7, respectively. A small loading volume for the wells may increase the error due to surface tension and evaporation. If not for the surface tension, the number of enantiomers on the light-path would be same regardless of evaporation. However, in the presence of both surface tension and evaporation, the number of enantiomers on the light-path would be different, resulting in an error in CD. We assume that this type of error can be reduced in a fully automated HTE run because of the fast and parallel process. Also, replacing acetonitrile (b.p. 82 °C) with less volatile propionitrile (b.p. 97 °C) could improve the accuracy.^53^

**Table 1:** Average absolute errors (% *ee*) were calculated from the respective *ee* values determined by a chiral HPLC and a CD microplate reader: 7.2 for 2 and 5.7 for 3

Samples for 2	% *ee* by HPLC	% *ee* by CD	Differences (% *ee*)	Samples for 3	% *ee* by HPLC	% *ee* by CD	Differences (% *ee*)
2-1	−67.6	−54.6	13	3-1	−68.8	−59.5	9.3
2-2	−61.0	−57.1	3.9	3-2	−60.4	−63.4	3.0
2-3	−53.4	−47.4	6.0	3-3	−51.2	−54.6	3.4
2-4	−40.4	−37.8	2.6	3-4	−37.0	−43.2	6.2
2-5	−32.2	−37.7	5.5	3-5	−29.8	−40.7	10.9
2-6	−17.8	−21.5	3.7	3-6	−16.0	−30.7	14.7
2-7	−9.6	−11.0	1.4	3-7	−7.0	−15.6	8.6
2-8	8.4	8.0	0.4	3-8	9.2	9.1	0.1
2-9	15.4	17.0	1.6	3-9	18.2	14.4	3.8
2-10	29.8	39.6	9.8	3-10	32.6	31.5	1.1
2-11	38.6	50.0	11.4	3-11	41.4	44.8	3.4
2-12	47.0	65.7	18.7	3-12	56.0	59.8	3.8
2-13	59.4	68.0	8.6	3-13	63.8	60.9	2.9
2-14	67.0	81.8	14.8	3-14	71.6	62.9	8.7

## Summary

High-throughput experimentation has become important for expediting drug discovery and improving enantiomeric purity in asymmetric synthesis. With recent advances in chiroptical sensing methods, high-throughput *ee* determination is possible with increasing numbers of chiral functional groups. Although C–N axially chiral scaffolds are commonly found in atropisomeric drugs, their *ee* determination has been dependent on conventional chromatographic methods that may not be ideal for a HTE platform. Herein, we presented a CD-based assay utilizing simple metal coordination chemistry, not requiring the synthesis of any receptor or assembly. The sample preparation process for *ee* analysis is straightforward despite the need for purification. The use of a multi-well CD plate reader allowed *ee* determination in 1–3 seconds per sample. Albeit the current accuracy in *ee* measurements using the assay would not meet high standards required in the quality control of the pharmaceutical industry, instead the assay would be useful at the development stage of drugs where a large number of analytical samples are generated for screening. Overall, the assay adopting coordination chemistry for enhancing CD was successfully employed for the HT *ee* determination of atropisomeric triazole derivatives. We anticipate that with the use of HT workstations with robotic process automation, the assay could be implemented faster and with even lower-levels of error, offering a cost- and time-saving alternative to HPLC.

## Data availability

General procedure, CD data collection parameters, titration study, sample preparation protocol for HT *ee* determination, thin layer chromatography analysis, additional CD spectra and chiral HPLC spectra are available in the ESI.†

## Author contributions

J. L. designed and performed the CD based assay. M. G., and S. C. carried out the synthesis and characterization. J. L. wrote the original draft and M. G. wrote the synthesis part. S. J. M. and E. V. A. supervised and acquired funding for the project. All authors contributed to the revision of the manuscript and approved the final version.

## Conflicts of interest

There are no conflicts to declare.

## Supplementary Material

SC-014-D3SC01559A-s001

## References

[cit1] Brooks W. H., Guida W. C., Daniel K. G. (2011). The significance of chirality in drug design and development. Curr. Top. Med. Chem..

[cit2] Hancu G., Modroiu A. (2022). Chiral switch: between therapeutical benefit and marketing strategy. Pharmaceuticals.

[cit3] Hassan D. S., Kariapper F. S., Lynch C. C., Wolf C. (2022). Accelerated Asymmetric Reaction Screening with Optical Assays. Synthesis.

[cit4] Davies I. W. (2019). The digitization of organic synthesis. Nature.

[cit5] Mennen S. M., Alhambra C., Allen C. L., Barberis M., Berritt S., Brandt T. A., Campbell A. D., Castañón J., Cherney A. H., Christensen M. (2019). The evolution of high-throughput experimentation in pharmaceutical development and perspectives on the future. Org. Process Res. Dev..

[cit6] Shcherbakova E. G., James T. D., Anzenbacher P. (2020). High-throughput assay for determining enantiomeric excess of chiral diols, amino alcohols, and amines and for direct asymmetric reaction screening. Nat. Protoc..

[cit7] Labuta J., Ishihara S., Šikorský T., Futera Z., Shundo A., Hanyková L., Burda J. V., Ariga K., Hill J. P. (2013). NMR spectroscopic detection of chirality and enantiopurity in referenced systems without formation of diastereomers. Nat. Commun..

[cit8] Chen X., Kang Y., Zeng S. (2018). Analysis of stereoisomers of chiral drug by mass spectrometry. Chirality.

[cit9] You L., Zha D., Anslyn E. V. (2015). Recent advances in supramolecular analytical chemistry using optical sensing. Chem. Rev..

[cit10] Kubo Y., Maeda S. y., Tokita S., Kubo M. (1996). Colorimetric chiral recognition by a molecular sensor. Nature.

[cit11] Hu M., Yuan Y.-X., Wang W., Li D.-M., Zhang H.-C., Wu B.-X., Liu M., Zheng Y.-S. (2020). Chiral recognition and enantiomer excess determination based on emission wavelength change of AIEgen rotor. Nat. Commun..

[cit12] Jo H. H., Lin C.-Y., Anslyn E. V. (2014). Rapid optical methods for enantiomeric excess analysis: from enantioselective indicator displacement assays to exciton-coupled circular dichroism. Acc. Chem. Res..

[cit13] Wolf C., Bentley K. W. (2013). Chirality sensing using stereodynamic probes with distinct electronic circular dichroism output. Chem. Soc. Rev..

[cit14] Pilicer S. L., Dragna J. M., Garland A., Welch C. J., Anslyn E. V., Wolf C. (2020). High-throughput determination of enantiopurity by microplate circular dichroism. J. Org. Chem..

[cit15] Herrera B. T., Pilicer S. L., Anslyn E. V., Joyce L. A., Wolf C. (2018). Optical analysis of reaction yield and enantiomeric excess: a new paradigm ready for prime time. J. Am. Chem. Soc..

[cit16] Shabbir S. H., Joyce L. A., da Cruz G. M., Lynch V. M., Sorey S., Anslyn E. V. (2009). Pattern-based recognition for the rapid determination of identity, concentration, and enantiomeric excess of subtly different threo diols. J. Am. Chem. Soc..

[cit17] Zhu L., Anslyn E. V. (2004). Facile quantification of enantiomeric excess and concentration with indicator-displacement assays: an example in the analyses of α-hydroxyacids. J. Am. Chem. Soc..

[cit18] Shcherbakova E. G., Brega V., Lynch V. M., James T. D., Anzenbacher Jr P. (2017). High-throughput assay
for enantiomeric excess determination in 1,2-and 1,3-diols and direct asymmetric reaction screening. Chem.–Eur. J..

[cit19] Thanzeel F. Y., Balaraman K., Wolf C. (2018). Click chemistry enables quantitative chiroptical sensing of chiral compounds in protic media and complex mixtures. Nat. Commun..

[cit20] Li B., Zhang J., Li L., Chen G. (2021). A rapid and sensitive method for chiroptical sensing of α-amino acids via click-like labeling with o-phthalaldehyde and p-toluenethiol. Chem. Sci..

[cit21] Prabodh A., Wang Y., Sinn S., Albertini P., Spies C., Spuling E., Yang L.-P., Jiang W., Bräse S., Biedermann F. (2021). Fluorescence detected circular dichroism (FDCD) for supramolecular host–guest complexes. Chem. Sci..

[cit22] Berova N., Di Bari L., Pescitelli G. (2007). Application of electronic circular dichroism in configurational and conformational analysis of organic compounds. Chem. Soc. Rev..

[cit23] Canary J. W., Allen C. S., Castagnetto J. M., Wang Y. (1995). Conformationally driven, propeller-like chirality in labile coordination complexes. J. Am. Chem. Soc..

[cit24] Cardenas M. M., Nguyen A. D., Brown Z. E., Heydari B. S., Heydari B. S., Vaidya S. D., Gustafson J. L. (2021). Atropisomerism as inspiration for new chemistry. Arkivoc.

[cit25] Oki M. (1983). Recent advances in atropisomerism. Top. Stereochem..

[cit26] Toenjes S. T., Gustafson J. L. (2018). Atropisomerism in medicinal chemistry: challenges and opportunities. Future Med. Chem..

[cit27] Glunz P. W. (2018). Recent encounters with atropisomerism in drug discovery. Bioorg. Med. Chem. Lett..

[cit28] H Zhou C., Wang Y. (2012). Recent researches in triazole compounds as medicinal drugs. Curr. Med. Chem..

[cit29] Aggarwal R., Sumran G. (2020). An insight on medicinal attributes of 1,2,4-triazoles. Eur. J. Med. Chem..

[cit30] Sugane T., Tobe T., Hamaguchi W., Shimada I., Maeno K., Miyata J., Suzuki T., Kimizuka T., Sakamoto S., Tsukamoto S.-i. (2013). Atropisomeric 4-phenyl-4H-1,2,4-triazoles as selective glycine transporter 1 inhibitors. J. Med. Chem..

[cit31] Wang J., Zeng W., Li S., Shen L., Gu Z., Zhang Y., Li J., Chen S., Jia X. (2017). Discovery and assessment of atropisomers of (±)-lesinurad. ACS Med. Chem. Lett..

[cit32] Waaler J., Leenders R. G., Sowa S. T., Alam Brinch S., Lycke M., Nieczypor P., Aertssen S., Murthy S., Galera-Prat A., Damen E. (2020). Preclinical lead optimization of a 1,2,4-triazole based tankyrase inhibitor. J. Med. Chem..

[cit33] Capelli A., Micheli F. (2001). UK-315716/UK-240455. Pfizer. Curr. Opin. Invest. Drugs.

[cit34] Wu Y.-J., Liao G., Shi B.-F. (2022). Stereoselective construction of atropisomers featuring a C–N chiral axis. Green Synthesis and Catalysis.

[cit35] Cheng J. K., Xiang S.-H., Li S., Ye L., Tan B. (2021). Recent advances in catalytic asymmetric construction of atropisomers. Chem. Rev..

[cit36] Yan J.-L., Maiti R., Ren S.-C., Tian W., Li T., Xu J., Mondal B., Jin Z., Chi Y. R. (2022). Carbene-catalyzed atroposelective synthesis of axially chiral styrenes. Nat. Commun..

[cit37] Zeng L., Li J., Cui S. (2022). Rhodium-Catalyzed Atroposelective Click Cycloaddition of Azides and Alkynes. Angew. Chem., Int. Ed..

[cit38] Prieto A. , Synthesis of N-Heterocycles via Intramolecular Pd-Catalyzed C-N Buchwald-Hartwig Reaction, in More Synthetic Approaches to Nonaromatic Nitrogen Heterocycles, 2022, vol. 1, pp. 307–331

[cit39] Petermayer C., Dube H. (2018). Circular dichroism photoswitching with a twist: axially chiral hemiindigo. J. Am. Chem. Soc..

[cit40] Choi S., Guo M. C., Coombs G. M., Miller S. J. (2023). Catalytic asymmetric synthesis of atropisomeric N-aryl 1,2,4-triazoles. J. Org. Chem..

[cit41] Kwon Y., Li J., Reid J. P., Crawford J. M., Jacob R., Sigman M. S., Troste F. D., Miller S. J. (2019). Disparate catalytic scaffolds for atroposelective cyclodehydration. J. Am. Chem. Soc..

[cit42] Canary J. W., Mortezaei S., Liang J. (2010). Transition metal-based chiroptical switches for nanoscale electronics and sensors. Coord. Chem. Rev..

[cit43] You L., Pescitelli G., Anslyn E. V., Di Bari L. (2012). An exciton-coupled circular dichroism protocol for the determination of identity, chirality, and enantiomeric excess of chiral secondary alcohols. J. Am. Chem. Soc..

[cit44] Berova N. , Nakanishi K. and Woody R. W., Circular dichroism: principles and applications, John Wiley & Sons, 2000

[cit45] Aromí G., Barrios L. A., Roubeau O., Gamez P. (2011). Triazoles and tetrazoles: prime ligands to generate remarkable coordination materials. Coord. Chem. Rev..

[cit46] Chen S.-S. (2016). The roles of imidazole ligands in coordination supramolecular systems. CrystEngComm.

[cit47] Mogensen S. B., Taylor M. K., Lee J.-W. (2020). Homocoupling Reactions of Azoles and Their Applications in Coordination Chemistry. Molecules.

[cit48] Andersson Trojer M., Movahedi A., Blanck H., Nydén M. (2013). Imidazole and triazole coordination chemistry for antifouling coatings. J. Chem..

[cit49] Dragna J. M., Pescitelli G., Tran L., Lynch V. M., Anslyn E. V., Di Bari L. (2012). In situ assembly of octahedral Fe(II) complexes for the enantiomeric excess determination of chiral amines using circular dichroism spectroscopy. J. Am. Chem. Soc..

[cit50] Althoff M. A., Bertsch A., Metzulat M. (2019). Automation of μ-SPE (Smart-SPE) and Liquid-Liquid Extraction Applied for the Analysis of Chemical Warfare Agents. Separations.

[cit51] Eerkes A., Shou W. Z., Naidong W. (2003). Liquid/liquid extraction using 96-well plate format in conjunction with hydrophilic interaction liquid chromatography-tandem mass spectrometry method for the analysis of fluconazole in human plasma. J. Pharm. Biomed. Anal..

[cit52] Rossi D. T., Zhang N. (2000). Automating solid-phase extraction: current aspects and future prospects. J. Chromatogr. A.

[cit53] Shcherbakova E. G., James T. D., Anzenbacher Jr P. (2020). High-throughput assay for determining enantiomeric excess of chiral diols, amino alcohols, and amines and for direct asymmetric reaction screening. Nat. Protoc..

