# Supplementary material for: High-throughput determination of enantiopurity in atroposelective synthesis of aryl triazoles
Source: Chem Sci. 2023 May 11;14(22):5992–9. doi: 10.1039/d3sc01559a (PMC10246677; doi:10.1039/d3sc01559a)
Supplement: SC-014-D3SC01559A-s001 [file SC-014-D3SC01559A-s001.pdf]

Electronic Supplementary Material (ESI) for Chemical Science.
This journal is © The Royal Society of Chemistry 2023

Supplementary Information for

High-Throughput Determination of Enantiopurity in Atroposelective Synthesis of Aryl Triazoles

Jongdoo Lim,† Melody Guo,‡ Sooyun Choi,‡ Scott J. Miller,*, ‡ and Eric V. Anslyn*,†

†Department of Chemistry, The University of Texas at Austin, Austin, Texas 78712, United States

‡Department of Chemistry, Yale University, New Haven, Connecticut 06520-8107, United States

Table of Contents

General procedure…..……………………………………………………………….….…………...….S2

CD data collection parameters……………………………..…………………………………………….S2

Titration study……………………...……………………………………...………………………S2

Sample preparation protocol for HT *ee* determination…..……………………...………….…………..S2

Thin layer chromatography analysis…..…………………..……………………...……………………S3

Additional CD spectra…………………………...……………………………...…………………S4

Chiral HPLC spectra…………………………………………………………………...……..……S7

References……………………..…………………………………………………….…..………..S11

General procedure

All reagents were purchased from commercial sources. Acetonitrile was spectrophotometric grade. All other solvents were ACS grade and used without further purification. Compounds 1, 2, and 3 were synthesized according to literature procedure.[s1] Compound characterization for these samples may be found in reference s1. Chiral HPLC data were acquired using an Agilent 1100 series analytical chiral HPLC equipped with a photodiode array detector (254 nm) with a Daicel Chiralpak IA column (5 µm particle size, 4.5 x 250 mm), flow rate of 1.0 mL/min, and either 18% EtOH/hexanes (for compound 2) or 20 % EtOH/hexanes (for compound 3). CD screening of the atropisomers **1**, **2**, and **3** were performed using either a Jasco J-815 spectropolarimeter in the Targeted Therapeutic Drug Discovery and Development Program facility at the University of Texas at Austin or an Ekko CD microplate reader manufactured by Hinds Instruments. Titration study was conducted by using a Cary 100 UV-Vis spectrophotometer from Agilent Technology. HyperSep™ silica cartridges (bed weight 50 mg, column capacity 1 mL) were used for a wash-elute step. All stock solutions for CD analysis were prepared using acetonitrile as the solvent: **1** (17.5 mM), **2** (17.5 mM), **3** (16 mM), and $Cu(OTf)_2$ (17.5 mM for complexation with **1** and **2**, 16 mM for complexation with **3**).

CD data collection parameters

Jasco J-815 with auto peltier 6-cell changer: Sample concentration in acetonitrile (1.75 mM for **1** and **2**, 1.6 mM for **3**), Type 21 macro cuvette with PTFE stopper (1 mm, UV quartz), sensitivity - standard, D.I.T. (integration time) - 1 sec, bandwidth - 1 nm, data pitch - 0.1 nm, scanning speed - 100 nm/min, accumulation - 2 times, temperature - measured at 20 ^{o}C

Ekko: Sample concentration in acetonitrile (1.75 mM for **2** and 1.6 mM for **3**), sample volume (40 µL per well), quartz microplate with lid and sealing foil (96 wells, 6.6 mm diameter, well capacity 300 µL, quartz glass, Hellma Analytics), 40 mdeg sensitivity, 2 s integration time, room temperature.

Titration study

The titration study was carried out using a Cary 100 UV-Vis spectrophotometer from Agilent Technology (10 mm cell, UV quartz). The spectra were obtained at 320 nm where only the complexes can absorb light. The concentration of copper(II) triflate in acetonitrile was set to 0.175 mM. The absorbance curve reached a plateau at approximately 3:1 ligand–metal molar ratio.

Sample preparation protocol for HT *ee* determination

Four atroposelective syntheses were performed using (*R*)-/(*S*)-TCYP as the catalyst: Two reactions for **2** (R_a and S_a) and two reactions for **3** (R_a and S_a), respectively. The two reaction solutions were mixed in the different ratios such as 19:1, 18:2, 17:3, 15:5, 14:6, 12:8, 11:9, 9:11, 8:12, 6:14, 5:15, 3:17, 2:18, and 1:19, affording a total of 14 samples. Calibration curves were constructed with the varied *ee* values of **2** (-78, -46, -23, 1, 25, 49, and 80%) and **3** (-82, -50, -26, -2, 22, 46, and 78%), respectively. A sample preparation protocol for CD analysis is as follows:

- A crude solution (0.5 mg/mL) in DCM (2 mL) was washed 2 times with 2 mL of 0.01 M HCl (aq).
- A solution (1mL) in DCM layer was loaded onto the silica cartridge.
- Wash the cartridge with either 1:3 EA:Hex (3 x 1mL) for **2** or 1:4 EA:Hex (4 x 1mL) for **3**.
- Elute the product with 7:1 DCM:MeOH (200 µL).
- Dry the sample with N_2-blowing.
- Dissolve the dried sample in acetonitrile (2mL) and determine the concentration by measuring the UV absorbance (241 nm) of solution (200 µL per well) with an Ekko microplate reader.

- A solution of copper(II) triflate in acetonitrile (3 eq.) was added to the solution.
- The concentration was adjusted to 1.75 mM for **2** and 1.6 mM for **3** by adding acetonitrile.
- A solution of the complex (40 μL) was added to a 96-well plate and analyzed by a CD microplate reader.

Thin layer chromatography analysis

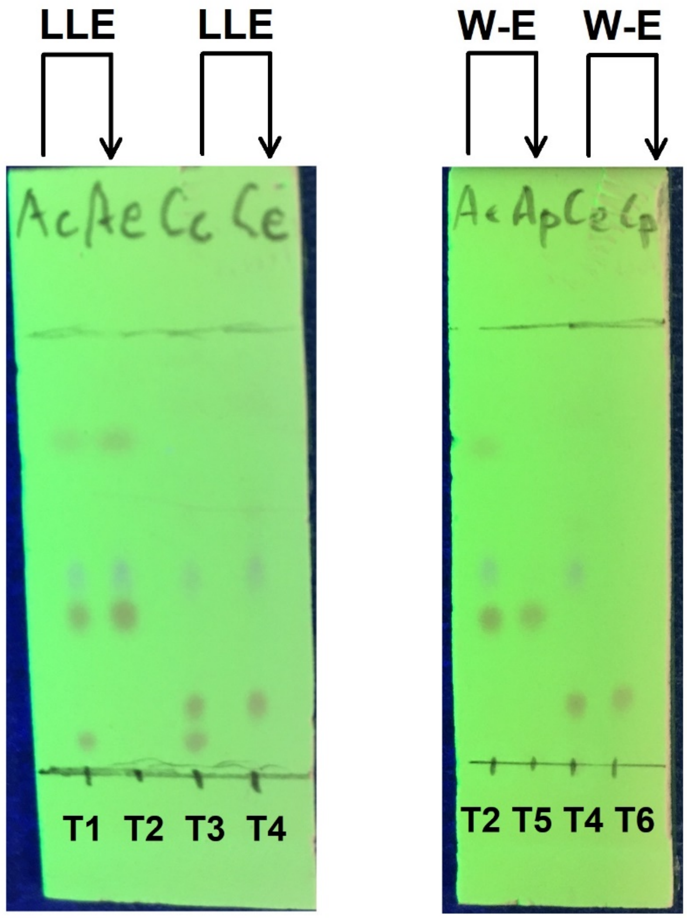

Figure S1. The sample purification process was monitored using thin layer chromatography (TLC). The TLC plates were developed in 1:1 EA:Hex: **T1** (the crude mixture of **3**), **T2** (the crude mixture **3** after liquid-liquid extraction (LLE)), **T3** (the crude mixture of **2**), **T4** (the crude mixture **2** after liquid-liquid extraction (LLE)), **T5** (the pure **3** after a wash-elute (W-E) step), **T6** (the pure **2** after a wash-elute (W-E) step).

Additional CD spectra

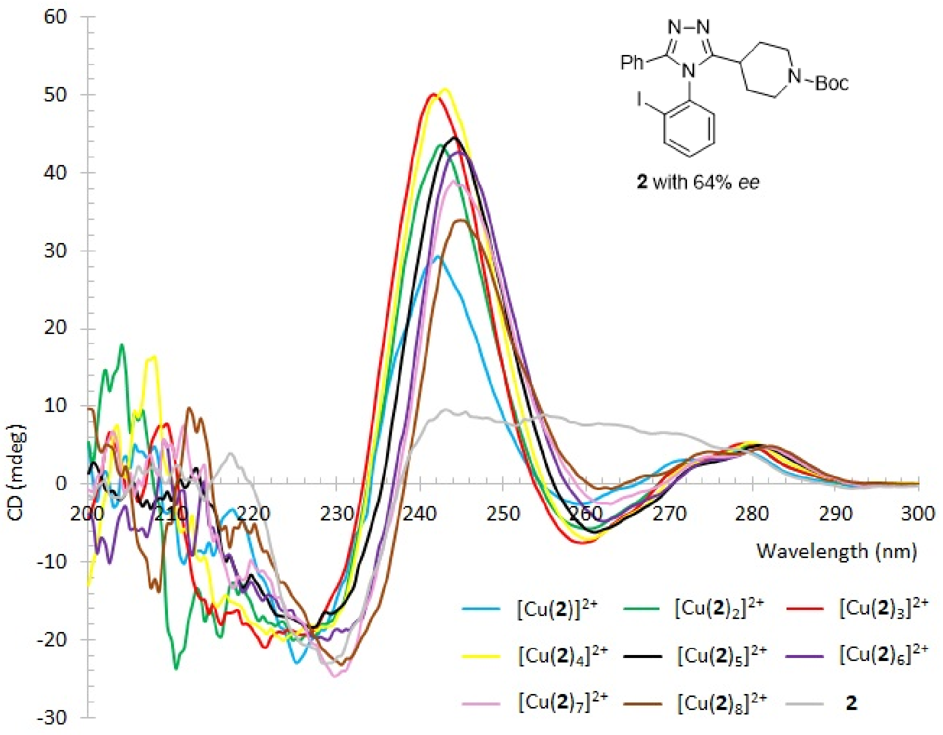

Figure S2. The CD spectra of **2** (64% *ee*) obtained with addition of copper(II) triflate (Jasco J-815, 1.75 mM in acetonitrile, 1 mm cell).

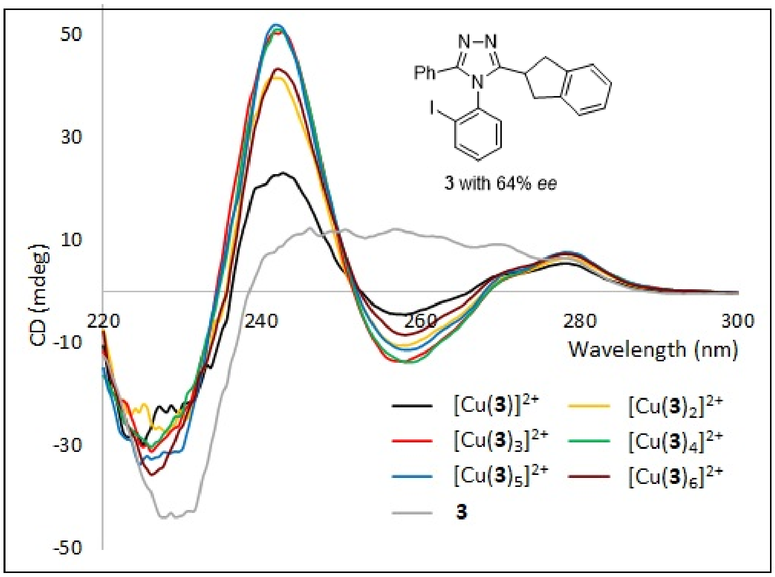

Figure S3. The CD spectra of **3** (64% *ee*) obtained with addition of copper(II) triflate (Jasco J-815, 1.6 mM in acetonitrile, 1 mm cell).

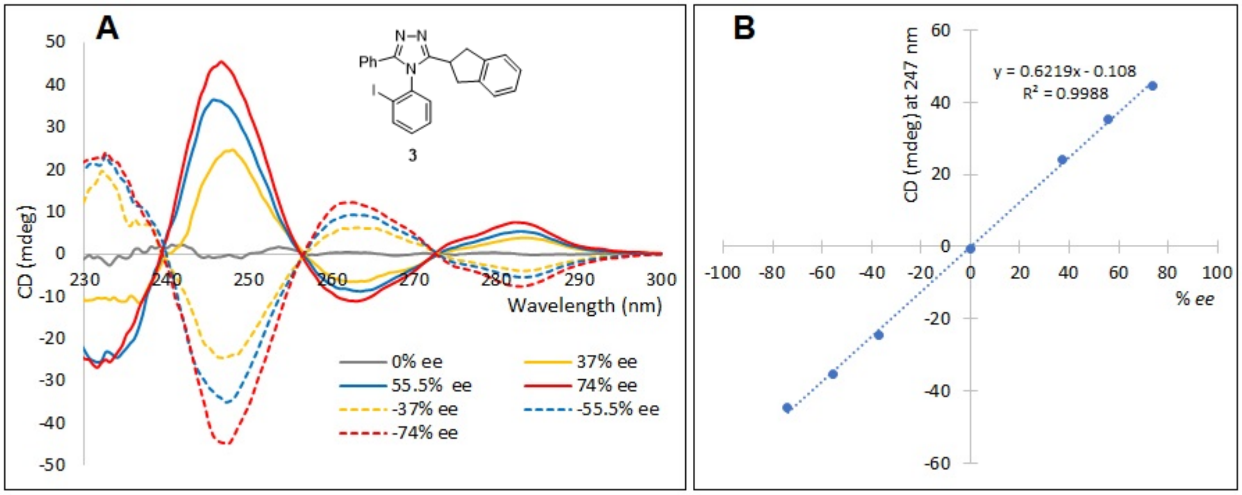

Figure S4. (A) CD spectra were recorded with $[Cu(\mathbf{3})_3]OTf_2$ having known *ee* values (Jasco J-815, 1.6 mM in acetonitrile, 1 mm cell). (B) The calibration curve was linear, exhibiting $R^2 = 0.9988$.

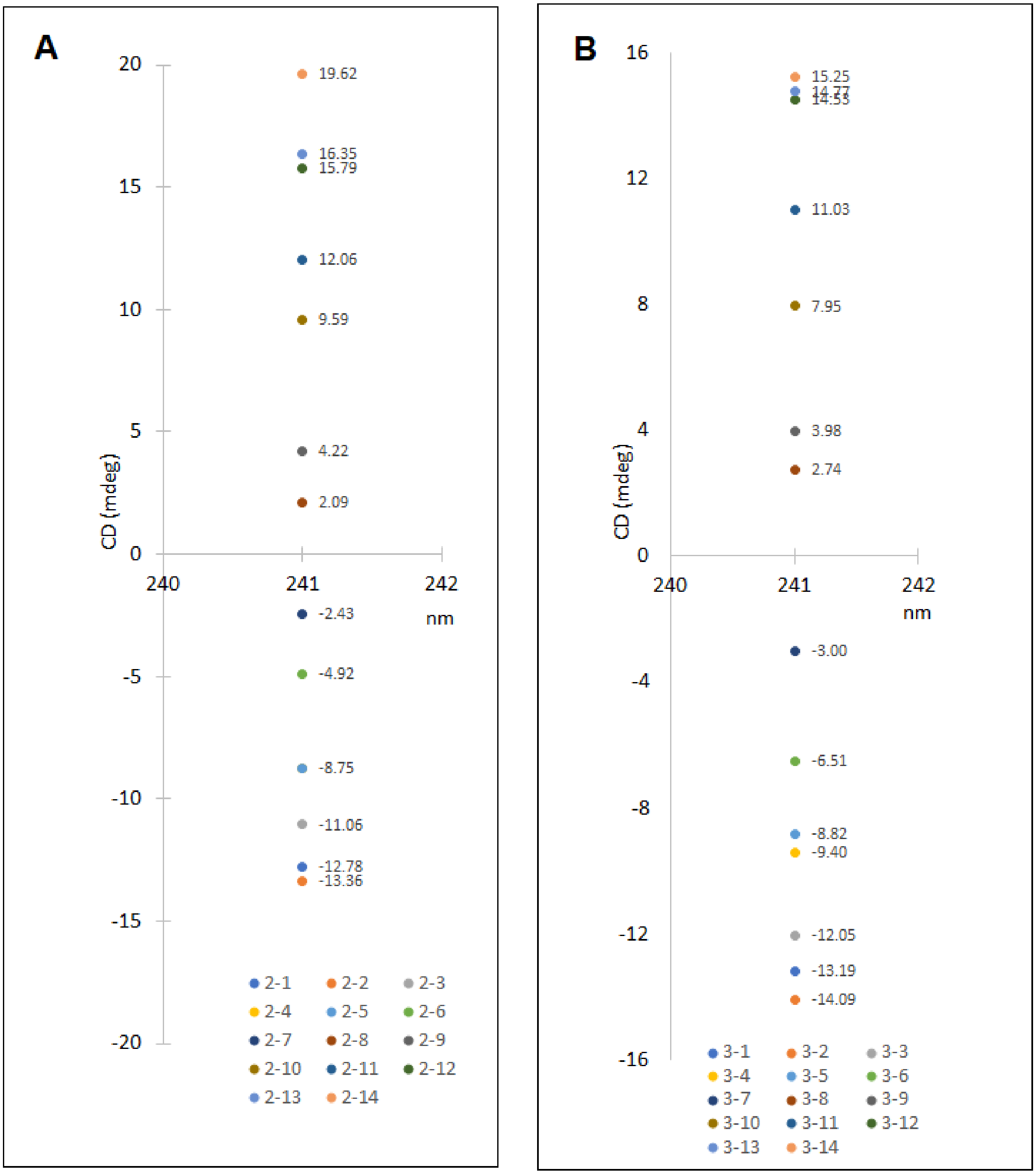

Figure S5. (A) CD spectra in the HT *ee* deterimiation of **2** (14 samples). (B) CD spectra in the HT *ee* deterimiation of **3** (14 samples).

Chiral HPLC spectra

Chiral HPLC spectra for the 14 samples of 2 (2-1 ~ 2-14)

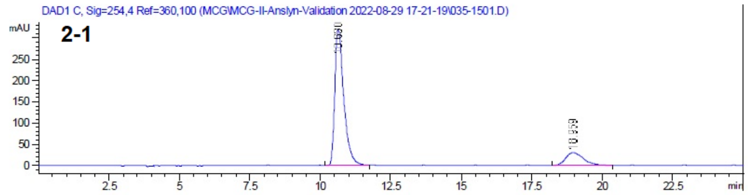

Signal 3: DAD1 C, Sig=254,4 Ref=360,100

Peak #	RetTime [min]	Type	Width [min]	Area [mAU*s]	Height [mAU]	Area %
1	10.630	VV	0.3364	7156.31348	320.60287	83.8279
2	18.959	VV	0.6447	1380.59912	30.46684	16.1721

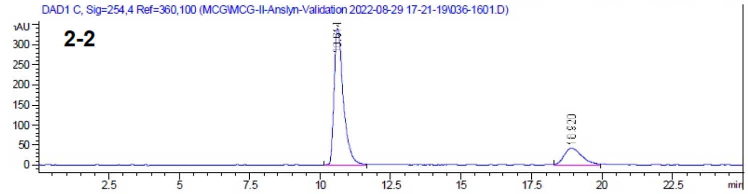

Signal 3: DAD1 C, Sig=254,4 Ref=360,100

Peak #	RetTime [min]	Type	Width [min]	Area [mAU*s]	Height [mAU]	Area %
1	10.614	VV	0.3433	7755.86182	341.07870	80.5476
2	18.920	VV	0.6507	1873.05420	42.12535	19.4524

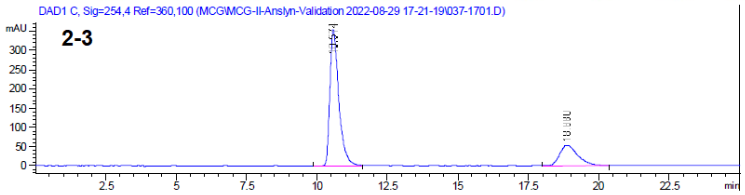

Signal 3: DAD1 C, Sig=254,4 Ref=360,100

Peak #	RetTime [min]	Type	Width [min]	Area [mAU*s]	Height [mAU]	Area %
1	10.574	VV	0.3306	7815.10889	355.30817	76.7090
2	18.880	VB	0.6651	2372.89111	53.12338	23.2910

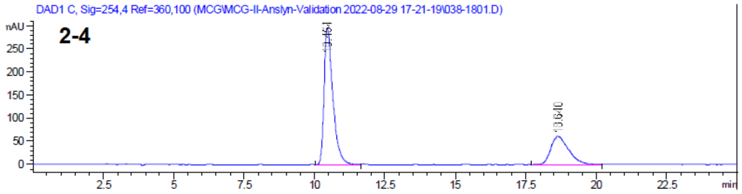

Signal 3: DAD1 C, Sig=254,4 Ref=360,100

Peak #	RetTime [min]	Type	Width [min]	Area [mAU*s]	Height [mAU]	Area %
1	10.454	VV	0.3296	6485.77295	296.06149	70.2052
2	18.640	VB	0.6563	2752.52905	61.25091	29.7948

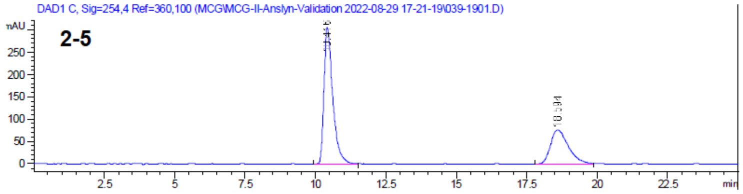

Signal 3: DAD1 C, Sig=254,4 Ref=360,100

Peak #	RetTime [min]	Type	Width [min]	Area [mAU*s]	Height [mAU]	Area %
1	10.416	VV	0.3288	6702.02832	306.84210	66.0801
2	18.594	VV	0.6654	3440.25171	77.27299	33.9199

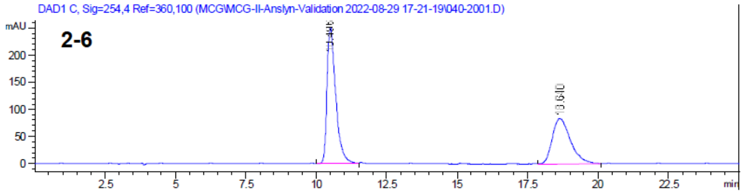

Signal 3: DAD1 C, Sig=254,4 Ref=360,100

Peak #	RetTime [min]	Type	Width [min]	Area [mAU*s]	Height [mAU]	Area %
1	10.496	VV	0.3285	5453.35986	252.01387	58.9103
2	18.640	VV	0.6858	3803.69043	83.75817	41.0897

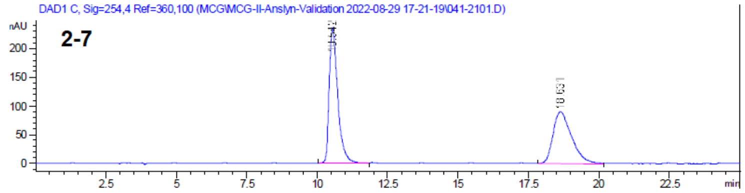

Signal 3: DAD1 C, Sig=254,4 Ref=360,100

Peak #	RetTime [min]	Type	Width [min]	Area [mAU*s]	Height [mAU]	Area %
1	10.542	VV	0.3187	5013.12939	237.05135	54.7529
2	18.631	VV	0.6776	4142.78955	90.24062	45.2471

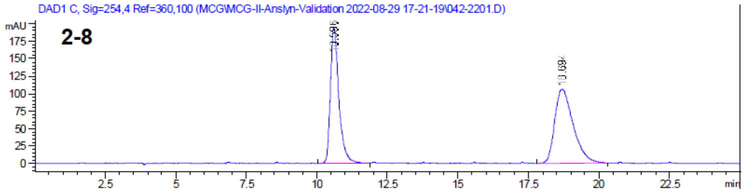

Signal 3: DAD1 C, Sig=254,4 Ref=360,100

Peak #	RetTime [min]	Type	Width [min]	Area [mAU*s]	Height [mAU]	Area %
1	10.596	VB	0.3126	4075.61206	194.39854	45.8098
2	18.694	VB	0.6715	4821.19727	106.22992	54.1902

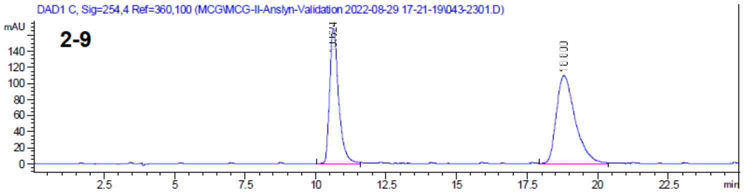

Signal 3: DAD1 C, Sig=254,4 Ref=360,100

Peak #	RetTime [min]	Type	Width [min]	Area [mAU*s]	Height [mAU]	Area %
1	10.624	VV	0.3359	3731.73120	168.81984	42.3044
2	18.800	VV	0.7072	5089.40430	109.71995	57.6956

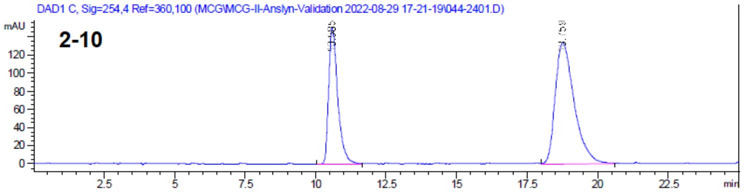

Signal 3: DAD1 C, Sig=254,4 Ref=360,100

Peak #	RetTime [min]	Type	Width [min]	Area [mAU*s]	Height [mAU]	Area %
1	10.585	VV	0.3374	3371.53174	150.50046	35.1043
2	18.759	VV	0.7143	6232.79053	133.59401	64.8957

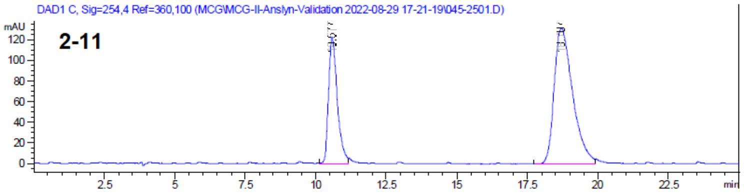

Signal 3: DAD1 C, Sig=254,4 Ref=360,100

Peak #	RetTime [min]	Type	Width [min]	Area [mAU*s]	Height [mAU]	Area %
1	10.577	VV	0.3281	2651.54883	122.70514	30.6872
2	18.707	VV	0.6901	5989.02490	131.81387	69.3128

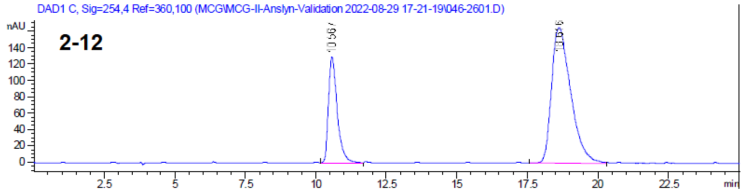

Signal 3: DAD1 C, Sig=254,4 Ref=360,100

Peak #	RetTime [min]	Type	Width [min]	Area [mAU*s]	Height [mAU]	Area %
1	10.567	VB	0.3271	2818.53369	129.93744	26.5113
2	18.616	VV	0.7039	7812.89063	165.75615	73.4887

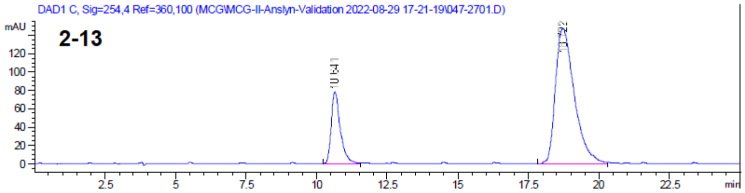

Signal 3: DAD1 C, Sig=254,4 Ref=360,100

Peak #	RetTime [min]	Type	Width [min]	Area [mAU*s]	Height [mAU]	Area %
1	10.641	VV	0.3386	1738.95117	77.85490	20.2768
2	18.722	BV	0.7068	6837.09326	147.50235	79.7232

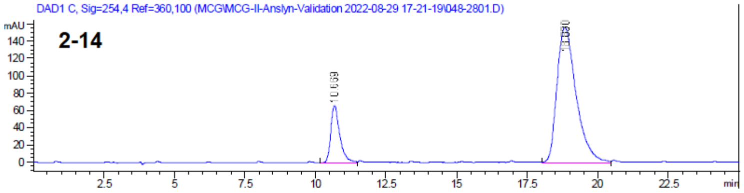

Signal 3: DAD1 C, Sig=254,4 Ref=360,100

Peak #	RetTime [min]	Type	Width [min]	Area [mAU*s]	Height [mAU]	Area %
1	10.669	BV	0.3288	1454.25061	65.56335	16.4900
2	18.830	VV	0.7211	7364.73340	156.48276	83.5100

Chiral HPLC spectra for the 14 samples of 3 (3-1 ~ 3-14)

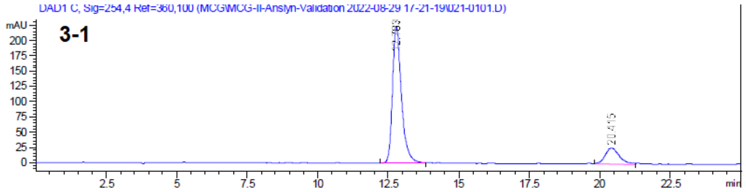

Signal 3: DAD1 C, Sig=254,4 Ref=360,100

Peak #	RetTime [min]	Type	Width [min]	Area [mAU*s]	Height [mAU]	Area %
1	12.783	VV	0.3326	4974.80762	224.47734	84.3558
2	20.415	VV	0.5270	922.60400	26.44824	15.6442

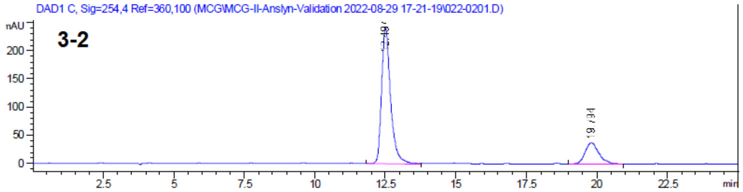

Signal 3: DAD1 C, Sig=254,4 Ref=360,100

Peak #	RetTime [min]	Type	Width [min]	Area [mAU*s]	Height [mAU]	Area %
1	12.497	VB	0.3327	5366.66064	241.99092	80.2191
2	19.794	VV	0.5102	1323.34253	38.00810	19.7809

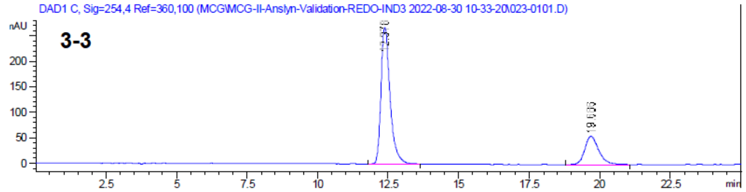

Signal 3: DAD1 C, Sig=254,4 Ref=360,100

Peak #	RetTime [min]	Type	Width [min]	Area [mAU*s]	Height [mAU]	Area %
1	12.378	VV	0.3370	6018.86816	269.01251	75.5999
2	19.686	VV	0.5311	1942.60852	55.95198	24.4001

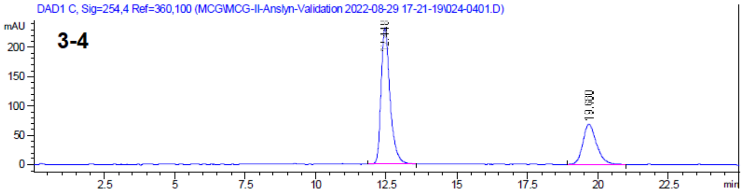

Signal 3: DAD1 C, Sig=254,4 Ref=360,100

Peak #	RetTime [min]	Type	Width [min]	Area [mAU*s]	Height [mAU]	Area %
1	12.448	BB	0.3302	5110.28271	232.70819	68.4748
2	19.680	VB	0.5161	2352.73145	68.60339	31.5252

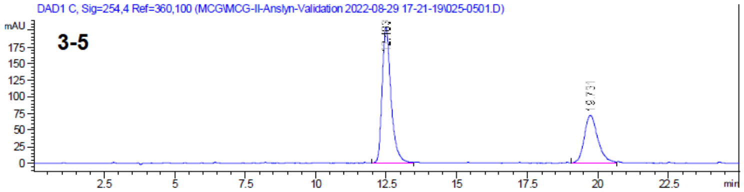

Signal 3: DAD1 C, Sig=254,4 Ref=360,100

Peak #	RetTime [min]	Type	Width [min]	Area [mAU*s]	Height [mAU]	Area %
1	12.483	VV	0.3360	4529.24658	204.79390	64.8512
2	19.731	VV	0.5128	2454.81421	72.54388	35.1488

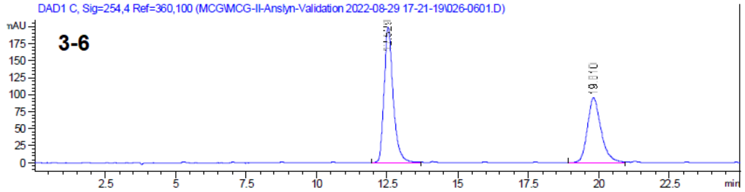

Signal 3: DAD1 C, Sig=254,4 Ref=360,100

Peak #	RetTime [min]	Type	Width [min]	Area [mAU*s]	Height [mAU]	Area %
1	12.529	VB	0.3348	4450.49268	199.09212	57.9989
2	19.810	VV	0.5062	3222.92041	95.38564	42.0011

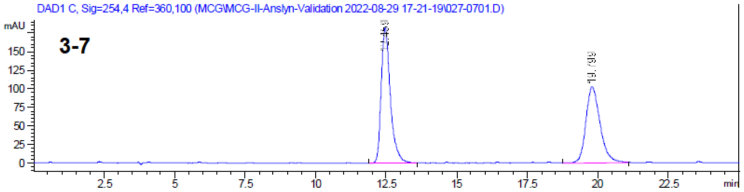

Signal 3: DAD1 C, Sig=254,4 Ref=360,100

Peak #	RetTime [min]	Type	Width [min]	Area [mAU*s]	Height [mAU]	Area %
1	12.459	VV	0.3398	4152.57324	183.62100	53.5099
2	19.799	VV	0.5347	3607.80298	102.99853	46.4901

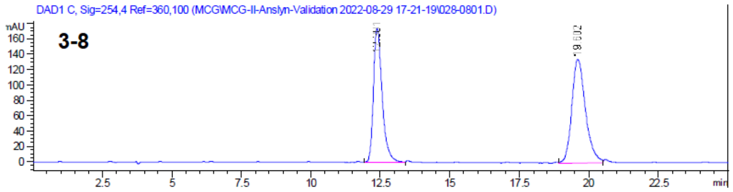

Signal 3: DAD1 C, Sig=254,4 Ref=360,100

Peak #	RetTime [min]	Type	Width [min]	Area [mAU*s]	Height [mAU]	Area %
1	12.381	VV	0.3297	3846.77954	175.50037	45.3656
2	19.602	VV	0.5133	4632.72168	135.37120	54.6344

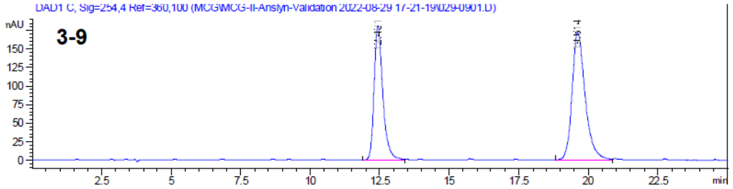

Signal 3: DAD1 C, Sig=254,4 Ref=360,100

Peak #	RetTime [min]	Type	Width [min]	Area [mAU*s]	Height [mAU]	Area %
1	12.431	VV	0.3254	3933.88086	181.08604	40.9219
2	19.614	VV	0.5017	5679.25195	172.69193	59.0781

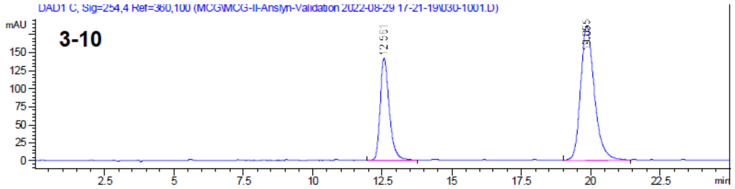

Signal 3: DAD1 C, Sig=254,4 Ref=360,100

Peak #	RetTime [min]	Type	Width [min]	Area [mAU*s]	Height [mAU]	Area %
1	12.561	VV	0.3514	3276.64014	141.91197	33.6548
2	19.855	VV	0.5243	6459.38770	186.37651	66.3452

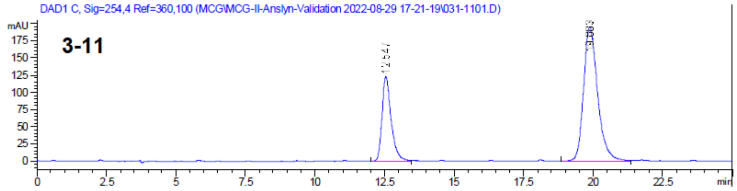

Signal 3: DAD1 C, Sig=254,4 Ref=360,100

Peak #	RetTime [min]	Type	Width [min]	Area [mAU*s]	Height [mAU]	Area %
1	12.547	BV	0.3456	2850.92065	123.35363	29.2665
2	19.883	VV	0.5381	6890.33545	195.05988	70.7335

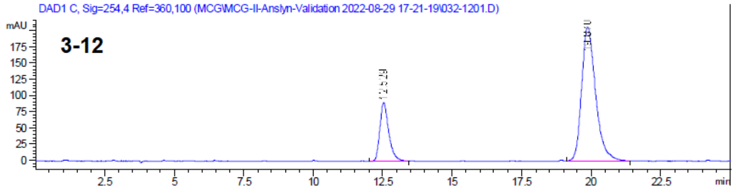

Signal 3: DAD1 C, Sig=254,4 Ref=360,100

Peak #	RetTime [min]	Type	Width [min]	Area [mAU*s]	Height [mAU]	Area %
1	12.529	VV	0.3438	2063.26953	90.57732	21.9686
2	19.870	VV	0.5347	7328.64893	207.15735	78.0314

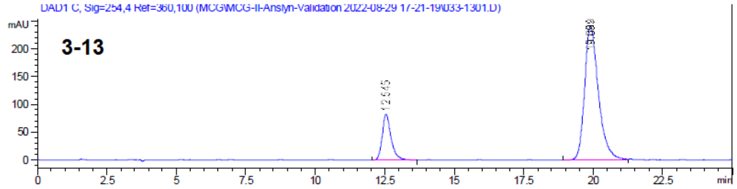

Signal 3: DAD1 C, Sig=254,4 Ref=360,100

Peak #	RetTime [min]	Type	Width [min]	Area [mAU*s]	Height [mAU]	Area %
1	12.545	VB	0.3382	1858.58228	82.68276	18.1155
2	19.899	VV	0.5242	8401.04199	241.30537	81.8845

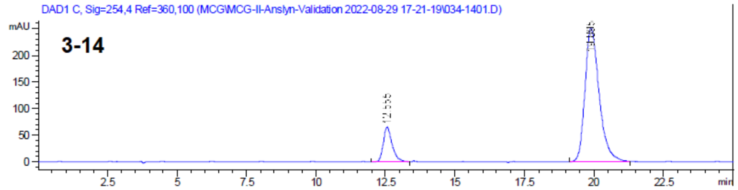

Signal 3: DAD1 C, Sig=254,4 Ref=360,100

Peak #	RetTime [min]	Type	Width [min]	Area [mAU*s]	Height [mAU]	Area %
1	12.555	VV	0.3442	1486.85327	65.16356	14.1697
2	19.895	VV	0.5425	9006.30371	252.24724	85.8303

References

s1. Choi, S.; Guo, M. C.; Coombs, G. M.; Miller, S. J., Catalytic Asymmetric Synthesis of Atropisomeric N-Aryl 1,2,4-Triazoles. *J. Org. Chem.* 2023, DOI: 10.1021/acs.joc.1022c02727.
